# Parvalbumin expressing interneurons control spike-phase coupling of hippocampal cells to theta oscillations

**DOI:** 10.1038/s41598-022-05004-5

**Published:** 2022-01-25

**Authors:** Michael Strüber, Jonas-Frederic Sauer, Marlene Bartos

**Affiliations:** 1 Present Address: Institute for Physiology I, University of Freiburg, Medical Faculty, Hermann-Herder-Str. 7, 79104 Freiburg, Germany; 2 Present Address: Kork Epilepsy Center, Landstraße 1, 77694 Kehl-Kork, Germany

**Keywords:** Neuroscience, Cellular neuroscience, Learning and memory, Neural circuits

## Abstract

Encoding of information by hippocampal neurons is defined by the number and the timing of action potentials generated relative to ongoing network oscillations in the theta (5–14 Hz), gamma (30–80 Hz) and ripple frequency range (150–200 Hz). The exact mechanisms underlying the temporal coupling of action potentials of hippocampal cells to the phase of rhythmic network activity are not fully understood. One critical determinant of action potential timing is synaptic inhibition provided by a complex network of Gamma-amino-hydroxy-butyric acid releasing (GABAergic) interneurons. Among the various GABAergic cell types, particularly Parvalbumin-expressing cells are powerful regulators of neuronal activity. Here we silenced Parvalbumin-expressing interneurons in hippocampal areas CA1 and the dentate gyrus in freely moving mice using the optogenetic silencing tool eNpHR to determine their influence on spike timing in principal cells. During optogenetic inhibition of Parvalbumin-expressing cells, local field potential recordings revealed no change in power or frequency of CA1 or dentate gyrus network oscillations. However, CA1 pyramidal neurons exhibited significantly reduced spike-phase coupling to CA1 theta, but not gamma or ripple oscillations. These data suggest that hippocampal Parvalbumin-expressing interneurons are particularly important for an intact theta-based temporal coding scheme of hippocampal principal cell populations.

## Introduction

In cortical circuits information is encoded and transmitted by action potential patterns of principal cells (PCs). For example, spatial information in the hippocampus is encoded by the number but also the exact timing of action potentials in relation to rhythmic field patterns^1,2^, which manifest as local field potential (LFP) oscillations. It is therefore assumed, that these network oscillations are important substrates for neuronal information processing^1–6^. In the rodent hippocampus, oscillatory network activity comprises mainly theta (~ 5 to 14 Hz), gamma (~ 30 to 80 Hz) and ripple oscillations (~ 150 to 200 Hz)^7^. Several studies have shown that rodent hippocampal PCs and interneurons (INs) discharge time locked to the phase of ongoing network oscillations in these different frequency ranges^8–10^. Indeed, *spike-phase coupling* has been observed in many brain circuits^9,11–14^. The mechanisms underlying spike-phase coupling are not fully understood. However, which PCs are active at which time point critically depends on the functional properties and synchrony of their excitatory and inhibitory synaptic inputs. Accordingly, in vivo whole-cell voltage-clamp recordings from hippocampal PCs showed that particularly inhibitory synaptic activity is temporally and spatially modulated by simultaneous neuronal network oscillations in different frequency ranges^15,16^. Synaptic inhibition is supplied by a complex network of predominantly Gamma-amino-hydroxy-butyric acid releasing (GABAergic) INs^9,17,18^. Among the many different types of GABAergic cells, perisoma-inhibiting Parvalbumin expressing INs (PVIs) have been shown to powerfully control the activity of their target cells^18–21^. In CA1, a large fraction of ripple-modulated inhibitory postsynaptic currents (IPSCs) seems to originate from presynaptic PVIs^15^. In a subgroup of place-coding CA1 pyramidal neurons, PVI-mediated inhibition seemed to change the theta oscillation phase of spiking^22^. However, the differential contribution of PVIs to the generation of hippocampal network oscillations and to phase coupling of principal neuron activity to the different frequency bands is not clear. Here, we addressed these questions by optogenetic silencing of PVIs in the hippocampal subfields CA1 and the dentate gyrus (DG) in freely moving mice.

## Results

**Figure 1:**
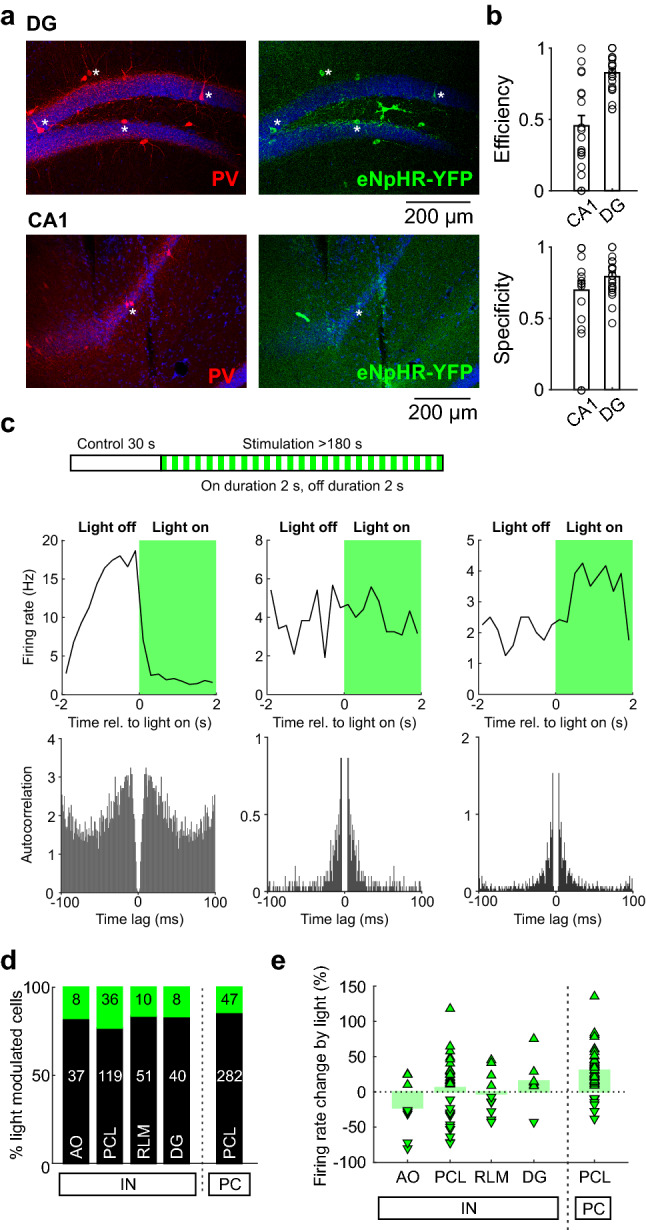
eNpHR-mediated silencing of hippocampal PV^+^ interneurons (PVIs). (**a**) Representative fluorescence microscopical pictures showing PV expression (red, left) and YFP infection (green, right) in DG (top) and CA1 (bottom). Note the vertical electrode tracks. White asterisks indicate neuronal somata. (**b)** Quantification of efficiency (top) and specificity (bottom) of PVI infection for four animals. Open circles represent individual brain slices, bars and error bars indicate mean and SEM. (**c)** Top, schematic illustrating the light stimulation protocol. After a 30 s control period with the light being switched off, 561 nm light is switched on and off alternately for ≥ 180 s with individual *light on* and *light off* periods of 2 s. Middle, average firing rate histograms aligned to the onset of light stimulation (at 0 ms time lag). Depicted are three different single units responding with a clear reduction in the activity (left), no firing rate change (middle) and an increasing activity level (right) during light stimulation (green area). Bottom, autocorrelograms of the corresponding single units shown on top. (**d**) Percentage of putative INs (left) and CA1 PCs (right) with either no change (black bars) or a significant change in firing rate in response to light stimulation (green section on top of the black bars). White numbers indicate the absolute numbers of neurons in the different anatomical regions (AO, alveus/oriens; PCL, pyramidal cell layer; RLM, radiatum/lacunosum-moleculare; DG, dentate gyrus). (**e**) Relative change in the firing upon light stimulation shown only for the significantly light-modulated single units.

To specifically and efficiently silence hippocampal PVIs, we injected adeno-associated viruses (AAVs) carrying a Cre-dependent genetic construct encoding for eNpHR-eYFP^23^ in the hippocampal subfields CA1 and DG of seven PV-Cre mice. Viral infection led to a specific expression of eNpHR in CA1 and DG PVIs (Fig. 1a, b; efficiency of infection DG 82.6 ± 3.0%, CA1 45.4 ± 7.4%; specificity of infection DG 79.3 ± 3.1%, CA1 69.6 ± 7.5%). To examine the effect of PVI silencing on the level of individual cells and neuronal populations, we implanted an optrode coupled to a microdrive (Supplementary Fig. S1) in the dorsal hippocampus and simultaneously recorded single units and the LFP at different radial layers ranging from CA1 *alveus/stratum oriens* (*AO*), *pyramidal cell layer* (*PCL*), *stratum radiatum/lacunosum-moleculare* (*RLM*) to the *DG* (see “Methods” section). Animals were allowed to freely explore their environment. After a baseline period of 30 s, we initiated a 561 nm-light stimulation protocol with alternating *light on*- and *light off* periods of 2 s duration. We determined the effect of local optogenetic PVI silencing by obtaining for every recorded single unit peri-stimulus firing rate histograms (Fig. 1c). We identified three response characteristics: Single units were either inhibited upon light delivery as would be expected from a direct eNpHR effect; excited, indicating a potential disinhibitory mechanism; or they did not show a significant change of their activity level (Fig. 1c). We recorded in total 638 putative individual cells located in the four hippocampal subareas AO, PCL, RLM and DG (Fig. 1d). On the basis of spike shape, autocorrelogram and baseline discharge rate, we could differentiate between putative INs and PCs (Fig. S2; “Methods” section)^8^. Outside the CA1 PCL, we identified only rarely putative PCs, further confirming the quality of our analysis method.

Light stimulation caused in 14.3% of putative CA1 PCs and in 20.1% of hippocampal INs a significant change in their discharge rate (Fig. 1d). As expected, the majority of these light sensitive CA1 PCs were disinhibited (Fig. 1e; Supplementary Fig. S3), resulting in the elevation of the average firing rate of the CA1 PC population to 110.8% (*p* = 2.34 × 10^–54^, paired t-test; see also Supplementary Fig. S4). In contrast, the responses of light sensitive putative INs depended on their layer-specific location. In AO, light-mediated silencing was the dominant response (Fig. 1e), while in CA1 PCL and RLM, INs were as likely to be inhibited as excited (Supplementary Figs. S3 and S4). In the DG, INs were predominantly excited (Fig. 1e). In summary, despite the high PVI connectivity with local PCs ^20^, most of the recorded cells did not show any significant change in their activity level during optogenetic PVI silencing. Moreover, the response characteristics were cell type- and layer-specific with CA1 PCs being largely disinhibited, while INs displayed heterogeneous responses (Supplementary Fig. S4). Among them, silenced INs most likely expressed eNpHR and corresponded to virally infected PVIs, while excited INs might be relieved from the optogenetically silenced perisomatic synaptic inhibition.

### Optogenetic silencing of hippocampal PVIs does not affect LFP oscillations

**Figure 2:**
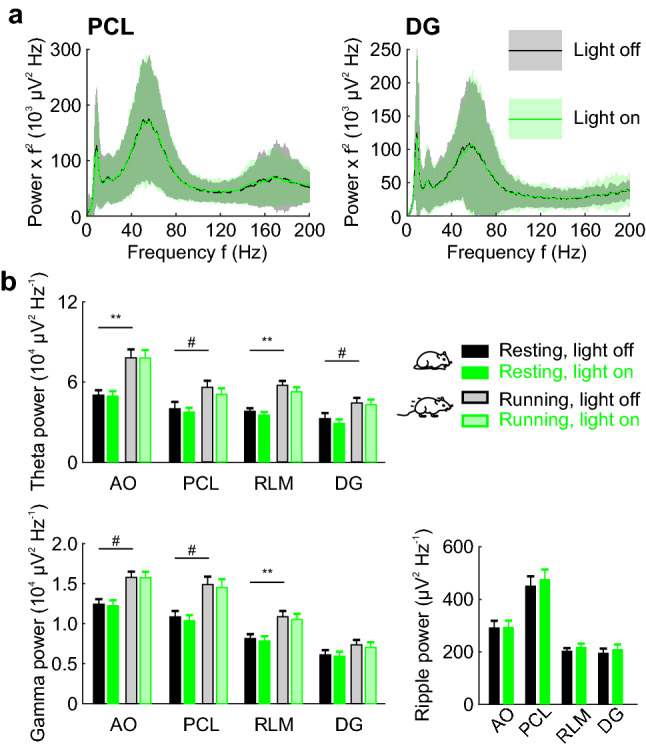
Optogenetic silencing of PVIs does not affect the power of hippocampal LFP oscillations. (**a**) Average power spectra of the LFP recorded in the CA1 PCL (left) or in the DG (right) during *light off* (black) or *light on* (green) periods. For illustrative purposes, power was multiplied by the squared frequency. Black and green lines are means of 136 (PCL) and 110 (DG) recordings in 7 animals, shaded areas indicate the respective standard deviations. (**b**) Total oscillation power in the theta (5–14 Hz, top), gamma (30–80 Hz, bottom left) and ripple (150–200 Hz, bottom right) frequency range during *light on* (green) and *light off* (black) periods. Episodes when the animal was sitting still (black and green bars) were analyzed separately from running episodes (grey and light green bars). Light-mediated silencing of PVIs did not lead to significant changes in the power of LFP oscillations. In contrast, during running theta and gamma power was significantly higher in different anatomical regions. Ripple events only occurred during resting periods of the animal. AO, alveus/oriens; PCL, pyramidal cell layer; RLM, radiatum/lacunosum-moleculare; DG, dentate gyrus.

PVIs are thought to be key players in the generation of neuronal network oscillations, particularly those at higher frequency ranges including gamma and ripple activity patterns^7,15,21,24–26^. To examine whether optogenetic PVI silencing may influence oscillatory activity we obtained average power spectra of LFP recordings from the four hippocampal subareas during *light on* and *light off* periods (Fig. 2a). Intriguingly, we observed no change in theta, gamma or ripple power during periods of PVI silencing compared to control periods (Fig. 2a; see also Supplementary Fig. S5 for an alternative PSD computation). Oscillation power and frequency is influenced by the animal’s movement^27–29^. We therefore compared theta and gamma power during *light on* and *light off* periods when animals were moving and resting (Fig. 2b). As expected^30,31^, discernible ripple oscillations were almost exclusively detected in the CA1 PCL during the resting period. Consistent with previous reports^27,32^, theta and gamma power was markedly higher during running than resting periods in all hippocampal subareas but the DG, where we found a non-significant trend towards higher gamma power during running (Fig. 2b). Nevertheless, optogenetic PVI silencing did not affect the power of theta, gamma or ripple oscillations irrespective of the animal’s behavioral state (Fig. 2b).

### Spike-phase coupling of putative CA1 PCs to theta oscillations but not gamma or ripple activity depends on intact PVI inhibition

**Figure 3:**
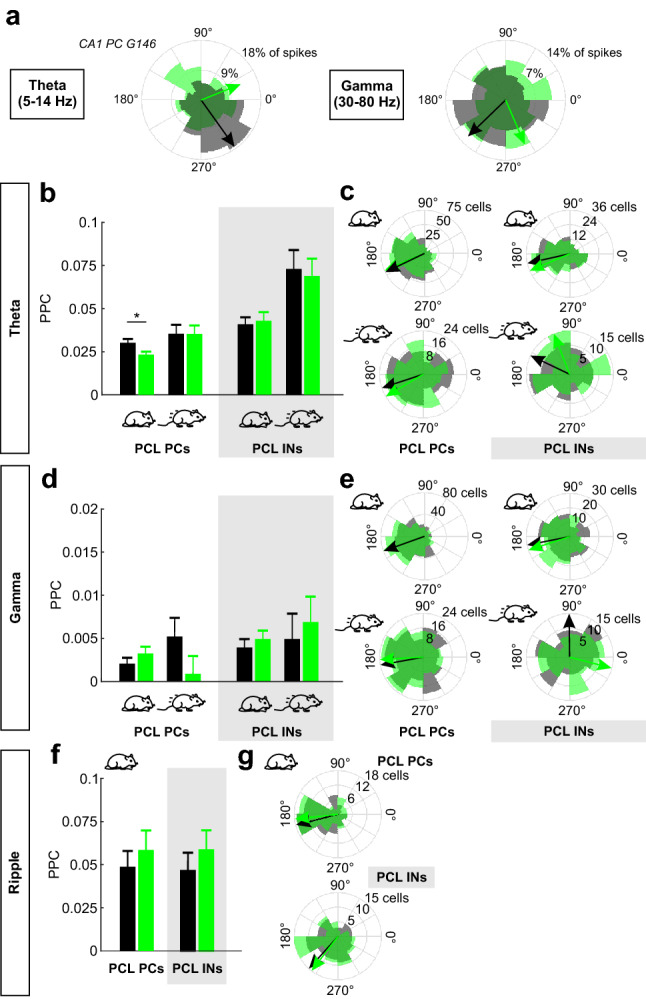
Optogenetic silencing of PVIs disrupts spike-phase coupling of CA1 PCs. (**a**) Polar phase histograms of an example CA1 PC showing significant spike-phase coupling with theta (left) and gamma (right) oscillations during control conditions (grey areas). During light illumination significant spike-phase coupling is lost (green areas). Arrows indicate preferred oscillatory phase of firing. Length of the arrows is adjusted to the maximum of the respective polar phase histograms. (**b**) Strength of spike-theta phase coupling of CA1 PCs (left) and PCL INs (right, grey box) quantified by the pairwise phase consistency (PPC) during *light on* (black) and *light off* (green) periods. Resting and running periods were analyzed separately (schematic mice below). (**c**) Polar histograms depicting the distribution of preferred theta oscillation phases for the following conditions: CA1 PCs, resting mice (top left); PCs, running mice (bottom left); PCL INs, resting (top right); INs, running (bottom right). Grey areas summarize *light off*-, green areas *light on* periods. Black/green arrows indicate average oscillation phase of spiking. Light-mediated PVI silencing did not affect the average oscillation phases of spiking. (**d**) and (**e**) In analogy to (**b**) and (**c**), analysis of spike-gamma phase coupling of CA1 PCs and PCL INs with respect to the strength (**d**) and the preferred gamma phase of spiking (**e**) in resting and running mice. (**f**)**,** (**g**) Analogous to (**b**) and (**c**), analysis of spike-ripple phase coupling of CA1 PCs and PCL INs regarding the strength of spike-ripple phase coupling (**f**) and preferred ripple phase of spiking (**g**) only for resting episodes. PCL, pyramidal cell layer. *p < 0.05.

Perisomatic PVI-mediated inhibition has been shown to critically determine the time point of action potential generation in postsynaptic cells^20,33^. Hippocampal principal neurons receive a large number of inhibitory perisomatic inputs^34,35^. We therefore asked how strong this PVI-mediated inhibition might contribute to temporal coupling of principal neuron spikes to the phases of theta, gamma and ripple cycles (i.e. spike-phase coupling). To address this question we focused on CA1 PCs due to the large data set collected in this area and the predominant expression of ripple oscillations in CA1. We obtained polar phase histograms for each PC during control and *light on* periods (see Supplementary Fig. S6 for example phase histograms of a random set of single units). The representative CA1 PC depicted in Fig. 3a exhibited clear spike-phase coupling to ongoing theta and gamma oscillations during control conditions. Significant spike-phase coupling was lost during *light on* periods (Fig. 3a; theta coupling: Rayleigh test for non-uniformity *p* = 4.34 × 10^–17^ for *light off versus p* = 0.991 *light on*; gamma coupling *p* = 9.33 × 10^–3^ *light off* and *p* = 0.844 *light on*). Since movement strongly affects the power of hippocampal network oscillations (Fig. 2b;^36–39^), we studied spike-phase coupling separately for resting and running periods. We quantified the coupling strength using pairwise phase consistency^40^ (PPC; see “Methods” section). During resting, optogenetic PVI silencing changed the spike-theta phase coupling in 29.3% of the recorded CA1 PCs, with 17.0% losing and 12.3% gaining significant spike-phase coupling during light application, resulting in a non-significant trend towards de-coupling of CA1 PC spiking from theta phases (Supplementary Table S1; *p* = 0.073, $\chi^{2}$ test). Moreover, the average coupling strength of CA1 PCs to theta phases was significantly reduced by optogenetic PVI silencing (Fig. 3b; PPC *light off* 0.03 ± 0.002 versus *light on* 0.023 ± 0.002; *p* = 0.013). The analysis of spike-phase coupling depends on the number of spikes included in the analysis. However, the number of CA1 PC spikes included in the spike-theta phase coupling analysis was not different for *light off* and *light on* periods (Supplementary Fig. S7).

During running periods, light-mediated PVI silencing changed the spike-theta phase coupling of 37.9% of the recorded CA1 PCs. However, the fraction of CA1 PCs losing spike-theta phase coupling was not different from the fraction of single units gaining spike-phase coupling (Supplementary Table S1; 18.1% losing versus 19.8% gaining spike-theta phase coupling, *p* = 0.684). Similarly, strength of theta phase-coupling was not different between *light off* and *light on* periods in running mice (Fig. 3b). In contrast to CA1 PCs, CA1 INs recorded in the PCL, rarely changed their spike-theta phase coupling in response to PVI silencing and their coupling strength was not affected by light stimulation during both resting and running periods (Fig. 3b; Supplementary Tab. S1). Importantly, the effect of PVI silencing on spike-theta phase coupling of individual PCs and INs could not be explained by light-mediated changes in their firing rate (Supplementary Fig. S8). In summary, PVI silencing reduces the strength of spike-theta phase coupling in CA1 PCs but not INs during resting periods.

While PVI silencing can change the strength of spike-theta phase coupling, the time point of PC or IN activity relative to the theta phase was not significantly different between *light off* and *light on* phases (Fig. 3d).

Next, we tested the influence of optogenetic PVI silencing on spike-phase coupling relative to gamma oscillations. During resting 18.4% and during running 15.2% of all individual CA1 PCs changed their spike-gamma phase coupling in response to PVI silencing (Supplementary Table S1), but on the level of the entire single unit population, PVI silencing did not alter the average strength or timing of spike-gamma phase coupling of CA1 PCs and PCL INs (Fig. 3e,f). Similarly, we observed that a substantial fraction of CA1 PCs and PCL INs responded to light stimulation with altered spike-ripple phase coupling. Indeed, 12.7% of CA1 PCs and 11.7% of PCL INs lost significant ripple phase coupling. Moreover, 13.9% of CA1 PCs and 16.9% of PCL INs gained significant ripple phase coupling upon PVI silencing (Supplementary Table 1). However, neither strength nor timing of CA1 PC- and PCL IN spike-ripple phase coupling were systematically affected by optogenetic PVI silencing (Fig. 3f, g).

In summary, optogenetic silencing of PVIs affected the spike-phase coupling of different fractions of hippocampal PCs and INs relative to theta, gamma and ripple activity. However, only spike-theta phase but not spike-gamma or -ripple phase coupling of CA1 PCs was systematically impaired during *light on* periods.

## Discussion

Here we show that optogenetic silencing of hippocampal PVIs in freely moving mice had no effect on the power and frequency of theta, gamma and ripple network activity patterns measured by LFP recordings (Fig. 2) but changed the temporal association of individual neuronal firing relative to the phases of these network oscillations in CA1 (Fig. 3; Supplementary Table 1):

First on the level of individual cells, many neurons with significant theta-, gamma- or ripple-phase coupling under control conditions lost their coupling during PVI silencing. Second, a fraction of those neurons which were not spike-phase coupled during control conditions gained significant spike-phase coupling during light stimulation. Finally on the population level, spike-phase coupling of CA1 PCs relative to theta but neither gamma nor ripple oscillations was systematically impaired in response to PVI silencing.

How can we explain this diversity in response characteristics of CA1 cells upon PVI silencing? Putative INs recorded in the PCL, corresponding most likely to PVIs^41^, remained either unaffected by light or responded to light stimulation with moderately reduced (by 44%) or enhanced discharge rate. INs showing silencing upon light delivery were most likely those PVIs expressing eNpHR (~ 45% of all CA1 PVIs, Fig. 1), whereas INs responding with elevated activity very likely were disinhibited from presynaptic PVI-mediated GABAergic signaling^24^. Such an inhomogeneously active PVI network with silenced, unaffected and even disinhibited INs could lead to the diverse effects observed in postsynaptic PCs. The heterogeneous reactions of PC activity to the light application are, moreover, amplified by the phenomenon of distance-dependence of perisomatic PVI-mediated inhibition onto hippocampal PCs, resulting in strong inhibition to spatially close synaptic partners but weak inhibition to more distant target cells^21,33^. Thus, strength of perisomatic inhibition can vary from PC to PC^42^ and optogenetic silencing of a fraction of PVIs could therefore cause no, mild or strong disinhibition of PCs—or even PC silencing in the situation of predominantly disinhibited presynaptic PVIs. We observed indeed that most light-modulated PCs responded with a firing rate increase to PVI silencing, while a small fraction showed reduced activity levels (Fig. 1e; Supplementary Fig. S4). Thus, CA1 PC populations were disinhibited upon PVI silencing, as would be expected from a strong PVI connectivity within the CA1 network.

Despite phase-decoupling of PC spikes from theta-oscillations and the described effects on the activity level in the hippocampal circuits, PVI silencing did not affect the power of the LFP signal. What could be the underlying reason? One explanation might be that the degree of PVI silencing or the population of silenced PVIs was too small to cause detectable changes in the LFP signal. Neuronal network simulations based on interconnected fast-spiking INs and PCs have shown that there is a marked dependence of network synchrony on the strength of feedback inhibition^24,43,44^. However, within a certain parameter space of inhibitory strength, coherent oscillations at gamma and ripple frequencies can still emerge^24,43,44^. Thus, a reduction in the strength of PVI-mediated inhibition within a given range may not necessarily lead to disruption of ongoing fast network oscillations. This hypothesis is in good agreement for example with previous work showing that PVI silencing only mildly affected the duration of ripple bouts and ripple LFP amplitude while it strongly reduced the peak amplitude of the ripple-associated inhibitory synaptic conductance recorded in CA1 PCs in vivo^15^. An alternative explanation might be that changes in the oscillation power in response to spatially restricted optogenetic manipulation of perisomatic inhibition might be local phenomena, which cannot be detected by single LFP recordings due to volume conduction but require electrode arrays and current source density analysis^21,45,46^.

In contrast to our observations related to theta oscillations, the average strength of PC spike-gamma and ripple phase coupling was not affected by PVI silencing. This result may be surprising as PVIs are strongly recruited during gamma and ripple oscillations^8–10,47^ and are thought to play essential roles in the generation of these activity patterns^15,48^. What might be the reason for the exclusive effect of PVI silencing on theta, but not gamma and ripple phase coupling of PC population activity? Since both gamma and ripple oscillations are more local network events^48,49^ than theta oscillations, we hypothesize that fewer PVIs are involved in the generation of gamma and ripple activity compared to theta oscillations. The moderate extent of the optogenetic silencing with ~ 45% of PVIs expressing eNpHR and being silenced to ~ 56% of their baseline firing rate might still allow the remaining PVI network to compensate for the silenced PVI fraction and to efficiently control PC spike timing relative to gamma and ripple phases. This compensatory action of the non-silenced interneuron population is further supported by our observation of increased firing rates in a subgroup of putative PCL interneurons during light stimulation periods (Supplementary Fig. S4). Moreover, PVI silencing indeed results in decoupling of a subset of PC activity from gamma and ripple oscillation phases, but this decoupled group of PCs is replaced by a different set of PCs gaining significant spike-gamma and -ripple phase coupling (Supplementary Table 1). These de-novo phase-coupled PCs might stand under predominant inhibitory control of PVIs which are either unaffected or even disinhibited during light stimulation periods. In contrast to the more local gamma and ripple activities, theta oscillations involve the entire hippocampus and a homogeneously active and intact PVI network might be necessary to guarantee effective coupling of PC activity to the theta oscillation phase.

In summary, our study provides evidence for the important role of perisomatic inhibition in controlling spike timing in hippocampal PCs, necessary for unperturbed encoding of information in cortical networks. Even a moderate reduction in the PVI activity and consequently the strength of PVI-mediated perisomatic inhibition in neuronal networks can have clear consequences on the activity level and spike timing of individual PCs and INs and thus, information processing employing spike-phase coupling^50^. We believe that these findings are of relevance for a better understanding of the circuit mechanisms that may underlie psychiatric diseases such as schizophrenia or Alzheimer’s disease, where particularly the network of PVIs seems to be strongly impaired ^43,51^.

## Methods

### Animals and surgery

Seven PV-Cre mice of either sex were used (B6;129P2-Pvalb^tm1(cre)Arbr^ /J mice; The Jackson Laboratory). At the time point of surgery, animals were 8–10 weeks of age and during recording 11–13 weeks. All animal procedures were performed in accordance with national and European legislation and institutional guidelines (University of Freiburg, Germany) and were approved by the Regierungspräsidium Freiburg (license number G15/106). The experiments were conducted in accordance with ARRIVE guidelines (ARRIVE 4 (randomization) and 5 (blinding) do not apply). Viral delivery and optodrive implantation were performed in one single surgery and targeted the dorsal hippocampus using stereotaxic coordinates (from bregma: anterior–posterior − 1.7 mm, medio-lateral 1.2 mm). At a depth of − 2.2 mm 1–2 µl of virus solution were delivered slowly over several minutes using a Hamilton syringe. The virus solution contained an adeno-associated-virus (AAV9) encoding eNpHR under a Cre-recombinase dependent promoter (~ 10^9^ viral genome copies per µl; pAAV–doublefloxed–eNpHR–EYFP–WPRE–pA; Addgene catalog #20949^23^; virus was provided by Dr. Lavinia Alberi by courtesy) dissolved in mannitol to a final concentration of ~ 7% mannitol to improve spatial spread of infection. The optodrive implant (see Supplementary Fig. S1) was introduced at the same site as the viral injection. The optrode bundle was lowered to − 1.1 to − 1.5 mm beneath dura mater. The implant was fixed to the skull by dental cement (C&B super bond, Sun Medical, Japan). Animals were allowed to recover for at least 14 days before recordings began and analgesia was provided with ibuprofen and buprenorphin.

### Recording and optical stimulation

Animals were allowed to move freely in different environments to stimulate exploratory behavior (open field, small home cage with bedding, large home cage with floor covered by paper tissue, platform without lateral walls). Electrical signals were recorded at a sampling frequency of 30,000 Hz using an Intan RHD2132 amplifier (Intan Technologies, USA) and the OpenEphys acquisition software (https://open-ephys.org). To induce optogenetic silencing, the recorded brain area was illuminated with green light of 561 nm wavelength emitted from a laser diode (LASOS Lasertechnik GmbH, Germany). Light intensity was adjusted to ~ 10 mW at the tip of the light guide.

### Quantification of the viral infection

To control the electrode implantation site and the viral infection quality, after finishing the recordings animals were perfused intracardially with 4% paraformaldehyde, brains were carefully removed and cut in 100 µm thick slices. Immunofluorescence labeling was performed as described earlier^43^ with a primary antibody against PV (rabbit anti-Parvalbumin, Swant; 1:1000), a secondary goat anti-rabbit Cy3 antibody (Jackson Immuno Research; 1:500) and the nuclear stain 4′,6-Diamidin-2-phenylindol (DAPI; 1:1000). Electrode implantation sites were identified and infection efficiency and specificity were quantified using an LSM710 Zeiss confocal microscope. Histological traces of electrode implantation showed that in all seven animals electrodes crossed the CA1 PCL. Furthermore, in six out of seven animals, electrode traces were additionally detected in the DG. Quality of viral infection was quantified in the DG and CA1 of those 2–6 slices corresponding to the experiment site (indicated by EYFP expression, electrode traces, superficial cortical wound resulting from virus injection) by counting all EYFP^+^|PV^+^, EYFP^+^|PV^-^ and EYFP^-^|PV^+^ cells within the area of interest (DG: complete DG section; CA1: up to ~ 600 µm lateral to the electrode lesions) and determining efficiency and specificity (Fig. 1b):

1 $$Efficiency = \frac{{Number ({\text{EYFP}} + |{\text{PV}} + )}}{{Number \left( {{\text{EYFP}} + |{\text{PV}} + } \right) + Number ({\text{EYFP}} - |{\text{PV}} + )}}$$

2 $$Specificity = \frac{{Number ({\text{EYFP}} + |{\text{PV}} + )}}{{Number \left( {{\text{EYFP}} + |{\text{PV}} + } \right) + Number({\text{EYFP}} + |{\text{PV}} - )}}$$

For image analysis and cell counting, Fiji based on ImageJ 1.49e and the in-built Cell Counter tool was used.

### Determination of the anatomical position of the recorded neurons

Throughout one experiment the optrode bundle was moved to different dorso-ventral depths corresponding to the following four areas from dorsal to ventral: CA1 alveus/stratum oriens (*AO*), CA1 pyramidal cell layer (*PCL*), CA1 stratum radiatum/ lacunosum-moleculare (*RLM*), and finally the dentate gyrus (*DG*). The allocation of the individual recording positions to one of those four brain areas relied on, first, the identification of the CA1 PCL by the large number of single units with principal cell spike phenotype and, second, the reconstruction of all other brain areas from the identified CA1 PCL based on a standard mouse brain anatomy (Allen mouse brain atlas): *PCL*-typical activity could be recorded on average over 200 ± 24 µm of vertical extent. Recordings obtained from a range of 200 µm directly dorsally to the dorsal border of the putative *PCL* were defined to be *AO* recordings. Recordings performed 400–1100 µm below the ventral border of *PCL* were defined as *DG* recordings. Data obtained between *PCL* and *DG* was allocated to the dendritic layers of *RLM*. Recordings obtained more dorsally than 200 µm above the dorsal *PCL* border or more ventrally than 1100 µm beneath the ventral *PCL* border were not included in the analysis. Isolated single units which could be recorded at different dorso-ventral positions were allocated to the brain region they most likely resided in. The results obtained from different depths were averaged.

### Local field potential (LFP) analysis

In a first step, LFP traces were manually inspected and obvious electrical artifacts were excluded from further analysis. In order to quantify oscillatory activity, LFP traces were subjected to multi-taper power spectrum density (PSD) estimation procedure using the Matlab function *mtspectrumc* included in the Chronux toolbox (*chronux.org*). For every multi-channel electrode, PSDs of the composing channels were averaged to obtain one representative PSD. To compare the oscillation profile for different experimental conditions (*light on* versus *light off*), LFP chunks of the respective condition were isolated from the original recording, stitched together and finally subjected to PSD analysis. Movement periods were defined as periods with momentary animal velocity > 1 cm s^-1^. Only those experiments were included in the LFP analysis, in which the animal moved ≥ 4 s (average period analyzed: 133.9 s, range 15.7–190.1 s (resting, *light off*); 87.5 s, range 11.8–120.5 s (resting, *light on*); 53.4 s, range 4.6–169.5 s (running, *light off*); 30.1 s, range 5.1–107.3 s (running, *light on*)). In Fig. 2b, to obtain total power in the theta, gamma and ripple frequency bands power densities at the respective frequencies from the corresponding power density estimation curves were summed and averaged.

To show robustness of our results against the exact methodology, we obtained power spectra in an alternative way employing Welch’s method using the Matlab function *pwelch* (Supplementary Fig. S5).

### Single unit analysis

#### Spike detection and sorting

Spike detection, clustering and subsequent manual re-sorting were performed using the Klusta Suite (https://klusta.readthedocs.io/en/latest/https://klusta.readthedocs.io/en/latest/^52^). After automatic spike detection and clustering, spike clusters were manually re-sorted using the KlustaViewa 0.3.0.beta1 graphical user interface into either single unit clusters or excluded from further analysis as putative artifacts. Manual sorting was based on visual inspection of the spike waveforms, on the autocorrelograms and on the distribution of the first three principal components.

#### Classification of single units into putative INs and PCs

Single units were classified as INs or PCs^8^ on the basis of (1) the width of the mean action potential waveform at 25% of the spike amplitude measured from baseline; (2) the first moment of the autocorrelogram in the time lag window of 0–50 ms, obtained using Matlab’s *xcorr* function with a temporal resolution of 1 ms; (3) the baseline firing rate during the control period of ≥ 30 s before light stimulation. For every single unit its percentile in the distribution of these three parameters was identified and used to determine a classifier coefficient for this respective single unit as follows:

3 $$Classifiercoefficient = \frac{1}{3} \times \left[ {Perc_{baseline rate} + Perc_{1st AC moment} + \left( {101 - Perc_{spike width} } \right)} \right]$$

Perc_baseline rate_, percentile for the respective single unit in the baseline firing rate distribution; Perc_1st AC moment_, percentile in the 1^st^ moment of the auto-correlogram distribution; Perc_spike width_, percentile in the spike width histogram.

The classifier coefficient ranges between 1 and 100. The lower the classifier coefficient, the more a single unit resembles an ideal principal neuron while higher values indicate a more IN-phenotype. All single units with a classifier coefficient ≥ 45 were regarded as putative INs. This border was chosen somewhat arbitrarily on the basis of the distribution of classifier coefficients suggesting a bimodal character (Supplementary Fig. S2).

#### Influence of optogenetic PVI silencing on the firing rate of individual units

To study the response of single unit firing to light induced silencing of PVIs, we obtained peri-stimulus firing rate histograms aligned to the time point of light ignition (Fig. 1c; Supplementary Fig. S3). The resulting histograms were averages of ≥ 45 individual firing rate plots for every single unit. To check whether single unit firing was significantly altered by light stimulation, a Wilcoxon signed-rank test was used comparing the average firing rate in the 2 s before to the 2 s during light stimulation.

#### Spike time coupling to the LFP

Temporal coupling of single unit activity to network oscillations was studied for the theta, gamma and ripple frequency range. First, for every spike time, the corresponding instantaneous phase of the theta, gamma and-if the spike occurred during a ripple phase—ripple oscillations was extracted from the Hilbert transform of the LFP after bandpass filtering the LFP in the respective frequency range^53^ (theta 5–14 Hz, gamma 30–80 Hz, ripple 150–200 Hz). Spike times of all identified single units were allocated to the four different experimental conditions “light off/resting”, “light off/running”, “light on/resting”, “light on/running”. For a single unit to be included in the comparison of two conditions, the number of action potentials allocated to each of those conditions had to be ≥ 10. A Rayleigh test for circular uniformity was used to check for significant coupling (*p* < 0.05). To quantify the strength of spike time coupling, the spike train to field pairwise phase consistency (*PPC*) measure was employed^40,53^. Differences in the preferred phase of firing between experimental conditions were statistically tested using a non-parametric multi-sample test for equal medians (*circ_cmtest* for Matlab)^54^.

### Data analysis and statistics

Data analysis and statistical testing were performed using Matlab R2017b (The Mathworks, USA). Values given in the text are mean ± standard error of the mean (SEM) if not indicated otherwise. To show the difference between two sets of data, two-sample t-test was used, if data were normally distributed. Normality was tested by the Lilliefors’ test using Matlab’s *lillietest* function. If the test for normality failed, Wilcoxon rank sum test was performed. To test the statistical difference in the frequency of categorical variables, $\chi^{2}$ test was used.

## Supplementary Information

Supplementary Information.
