# Supplementary material for: Parvalbumin expressing interneurons control spike-phase coupling of hippocampal cells to theta oscillations
Source: Sci Rep. 2022 Jan 25;12:1362. doi: 10.1038/s41598-022-05004-5 (PMC8789780; doi:10.1038/s41598-022-05004-5)
Supplement: Supplementary file 1 — Supplementary Information. [file 41598_2022_5004_MOESM1_ESM.pdf]

Parvalbumin expressing interneurons control spike-phase coupling of hippocampal cells to theta oscillations

Michael Strüber, Jonas-Frederic Sauer and Marlene Bartos

Supplementary Material

Supplementary Table

Units coupling during:		Theta				Gamma				Ripple	
		PCL PCs	PCL INs	PCL PCs	PCL INs	PCL PCs	PCL INs	PCL PCs	PCL INs	PCL PCs	PCL INs
+	+	50.1	73.3	23.7	49.5	7.2	18.6	1.7	5.7	17.7	19.5
+	-	17.0	7.6	18.1	11.4	8.1	11.6	7.9	8.6	12.7	11.7
-	+	12.3	6.4	19.8	15.2	10.3	14.0	7.3	11.4	13.9	16.9
-	-	20.6	12.8	38.4	23.8	74.4	55.8	83.1	74.3	55.7	51.9
p +|- *vs* -|+		0.073	0.672	0.684	0.417	0.301	0.519	0.841	0.490	0.815	0.357

Supplementary Tab. 1 | Fraction of CA1 PCs and PCL INs keeping or changing their spike-phase coupling during optogenetic PVI silencing. Left, schematic summarizing the four different spike-phase coupling options: single units could be significantly spike-phase coupled to (+) or uncoupled from (-) theta, gamma or ripple oscillations phases during *light off* periods. During light stimulation, the previously coupled units could either remain coupled (dark green; +|+) or be decoupled by the light stimulation (dark grey; +|-). Likewise, those units being uncoupled under control conditions could remain uncoupled during *light on* periods (light grey; -|-) or gain significant spike-phase coupling (light green; -|+). The table reports the fractions of these four categories for theta, gamma and ripple oscillations during resting (sitting mouse) or running periods (running mouse). Numbers indicate percentages. At the bottom of every column the *p* value of a χ^2 test is given, comparing the fraction of units losing significant spike-phase coupling with the fraction of units gaining significant spike-phase coupling in response to light stimulation.

Supplementary Figures

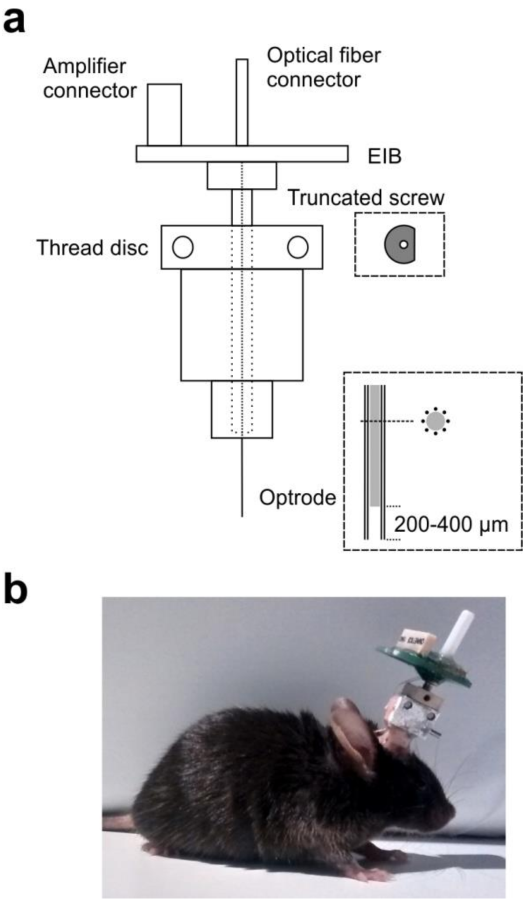

Supplementary Fig. S1 | a The design of the microdrive carrying an optrode consisting of one central optical fiber (diameter of 200 µm, Thorlabs) and up to 8 multi-channel electrodes (2-4 channels, tungsten tetrode wire, The Fine Wire Company, USA) glued to the centrally positioned optical fiber and protruding about 200-400 µm was based on the optrode drive described by (Anikeeva et al., 2011). Drive body and mechanical parts were custom made by the institute’s in-house workshop (H.-J. Weber and colleagues). Electrodes were gold-plated to reach an impedance of 0.03-0.33 MΩ (nanoZ Kit, Neuralynx, Germany). **b** Mice carrying the microdrive implant could move freely.

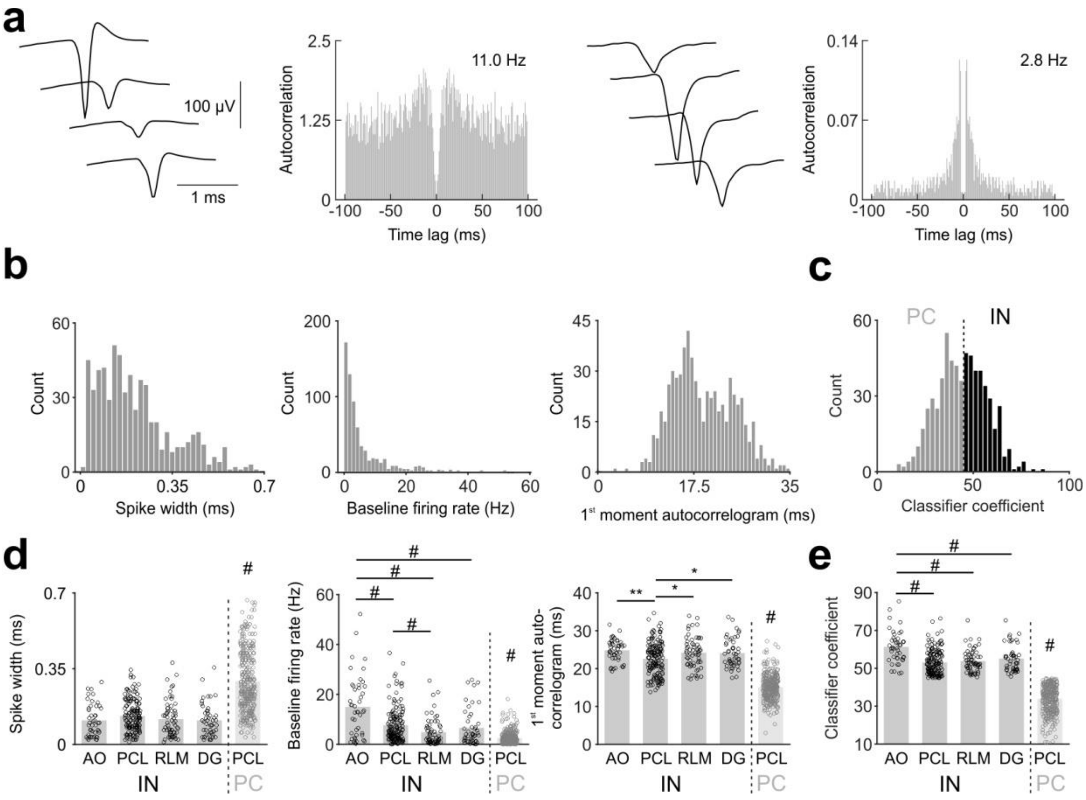

Supplementary Fig. S2 | Classification of single units in putative INs and PCs. a Two example single units recorded by the same electrode in the same recording session. Left, average spike waveforms recorded on the four different tetrode channels and the autocorrelogram of the corresponding single unit activity. This cell is active at 11.0 Hz during the control period, is characterized by a rather short spike duration and exhibits no strong tendency to discharge in bursts corresponding to a larger first moment of the autocorrelogram. Right, in analogy to the left single unit, average spike waveforms and autocorrelogram of a second cell are depicted. The second cell generates wider spikes, is active at lower rates (2.8 Hz) and exhibits a strong tendency to be active in bursts corresponding to a small first moment of the autocorrelogram. **b** Histograms showing (from left to right) the spike widths, baseline firing rates and first moments of the autocorrelogram. **c** Histogram showing the classifier coefficients based on the parameters depicted in **b** (see Methods) for all recorded single units. Note the bimodal shape of the classifier coefficient histogram. Single units with classifier coefficients ≥45 were defined as putative INs (black), putative PCs had classifier coefficients <45 (grey). Dotted vertical line illustrates the border between PCs and INs. **d,e** Comparison of the single unit classification parameters spike width (**d**, left), baseline firing rate (**d**, middle), first moment of the autocorrelogram (**d**, right) and the classifier coefficients (**e**) between the INs recorded in different hippocampal areas and CA1 PCs. Circles represent individual cells, bars indicate means. With respect to all parameters, PCs were significantly different from each IN subgroup. AO, alveus/stratum oriens; PCL, CA1 pyramidal cell layer; RLM, CA1 stratum radiatum/lacunosum-moleculare; DG, dentate gyrus. *, $p<0.05$; **, $p<0.01$; #, $p<0.001$ (Wilcoxon rank sum test).

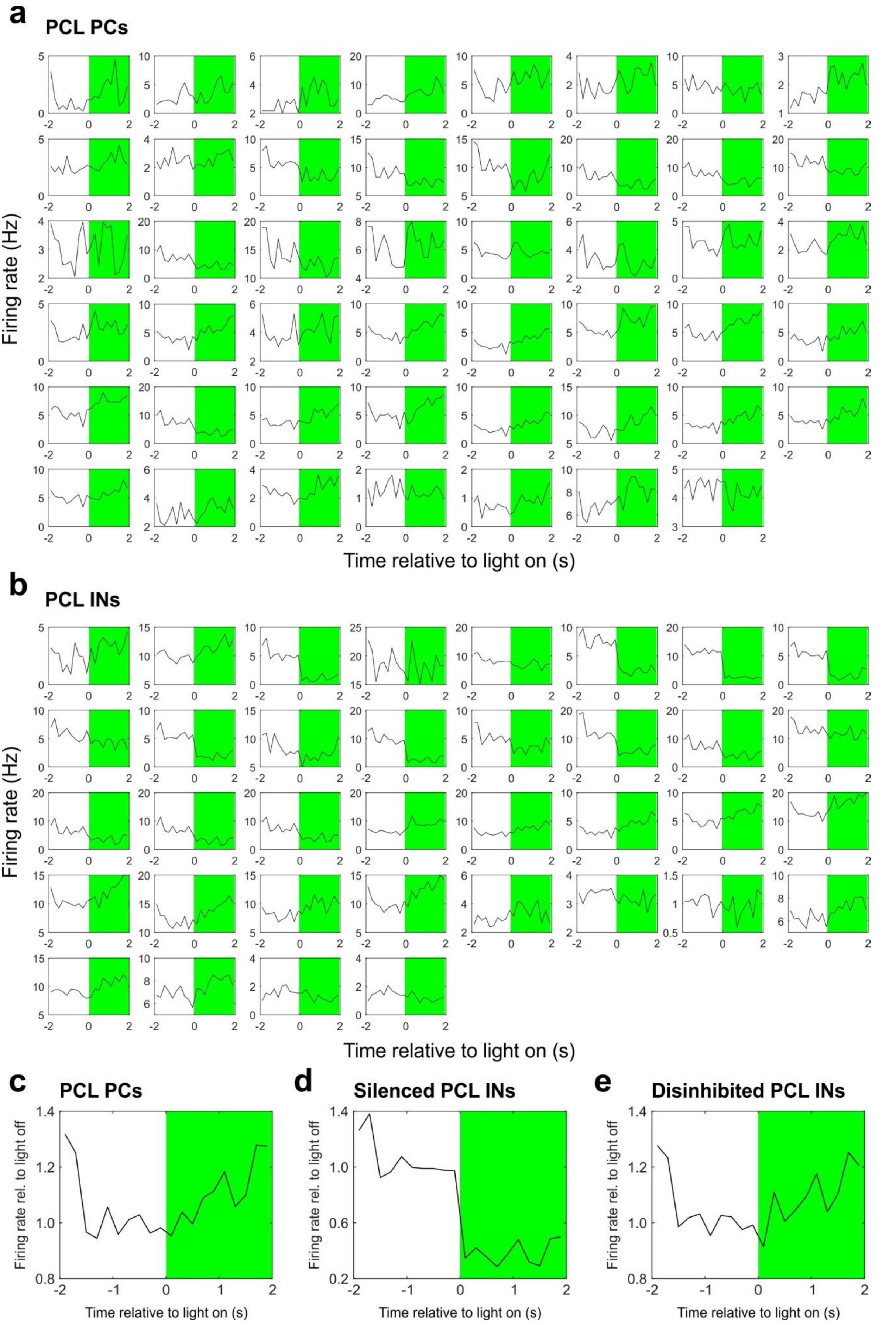

Supplementary Fig. S3 | Extent and time course of light-mediated firing rate change in individual single units. a, b Peri-stimulus firing rate histograms of CA1 PCs (**a**) and PCL INs (**b**) responding with a significant firing

rate change during light stimulation. Firing rate histograms are averages of ≥45 individual trials temporally aligned to the onset of the 2 s lasting light stimulation period (green background). **c-e** Average firing rate histograms aligned to the beginning of light stimulation (0 s) for all PCL PCs (**c**; N = 47 units), all PCL INs exhibiting significant light-mediated inhibition (**d**; N = 12 units) and all PCL INs exhibiting a significant increase in their firing rate during light stimulation (**e**; N = 24 units). Firing rate is normalized to the mean firing rate in the period -1.2 to 0 s before light stimulation onset. Note the difference in the time course with an immediate silencing effect of INs (**d**) but a delayed disinhibition effect of both PCs and INs (**c, e**).

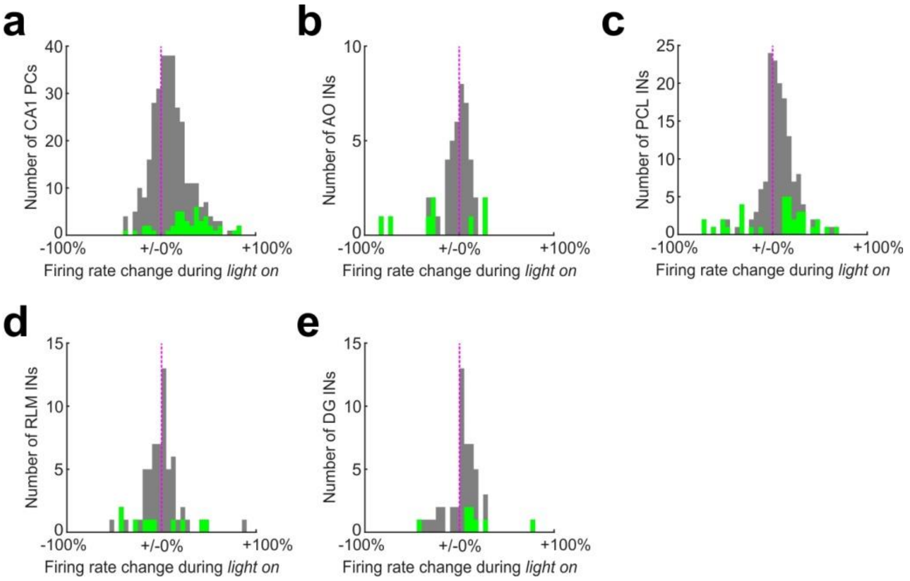

Supplementary Fig. S4 | Firing rate changes of different single unit types in response to optogenetic silencing of PVIs. Histograms summarizing the firing rate change in all recorded CA1 PCs (**a**), AO INs (**b**), PCL INs (**c**), RLM INs (**d**) and DG INs (**e**). Grey bars summarize all neurons without significant firing rate changes. Green bars indicate those cells showing significant changes of their firing rate during *light on* periods. Vertical purple lines indicate the condition of no firing rate change during PVI silencing.

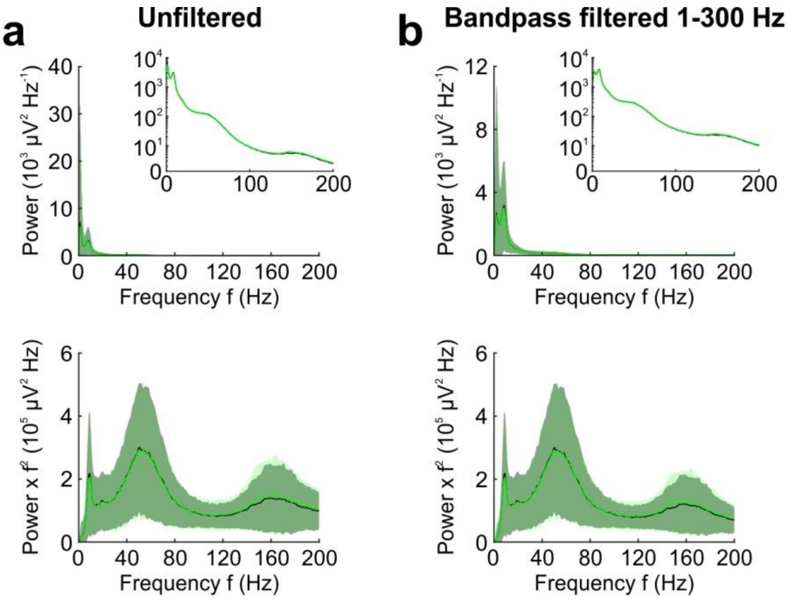

Supplementary Fig. S5 | Alternative power spectrum determination using Welch's method. a, b Mean power spectra of the raw (**a**) or the 1-300 Hz bandpass-filtered (**b**) LFP recorded in the CA1 PCL during *light off* (black) and *light on* periods (green). Shaded areas indicate the standard deviation. Power spectra are depicted raw (top) or multiplied with the squared frequency (bottom) to improve visibility of the higher frequency range. Inset on top, identical power spectra as in **a** and **b** top, but with logarithmic y-axis.

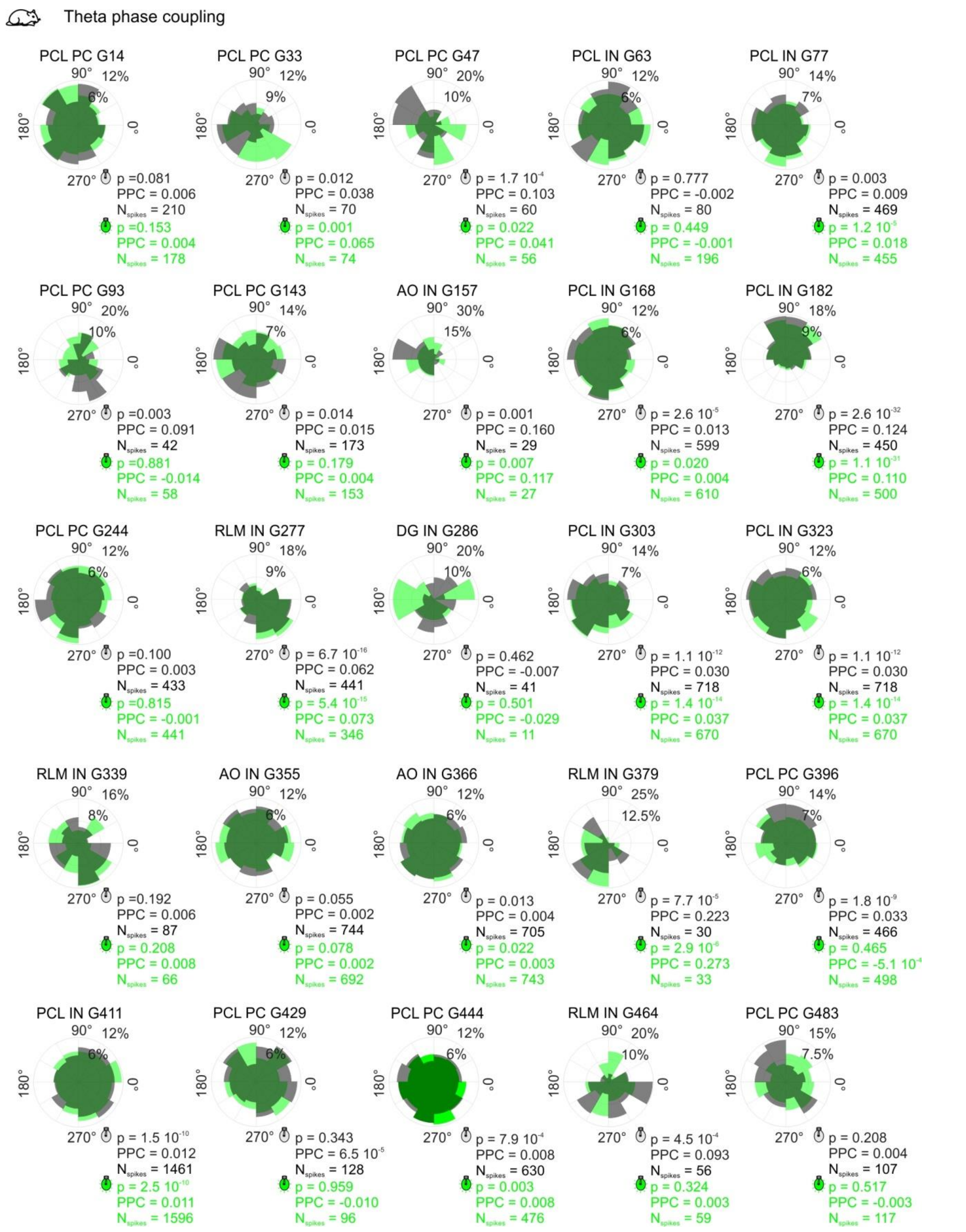

Supplementary Fig. S6 | Polar phase histograms of 25 randomly picked single units. Polar phase histograms summarize the theta phases at the time point of spiking during *light off* (grey) and *light on* periods (green) in resting

mice. Below each phase plot, the p-value of Rayleigh test, PPC and the number of spikes included in the analysis are listed for *light off* and *light on* periods.

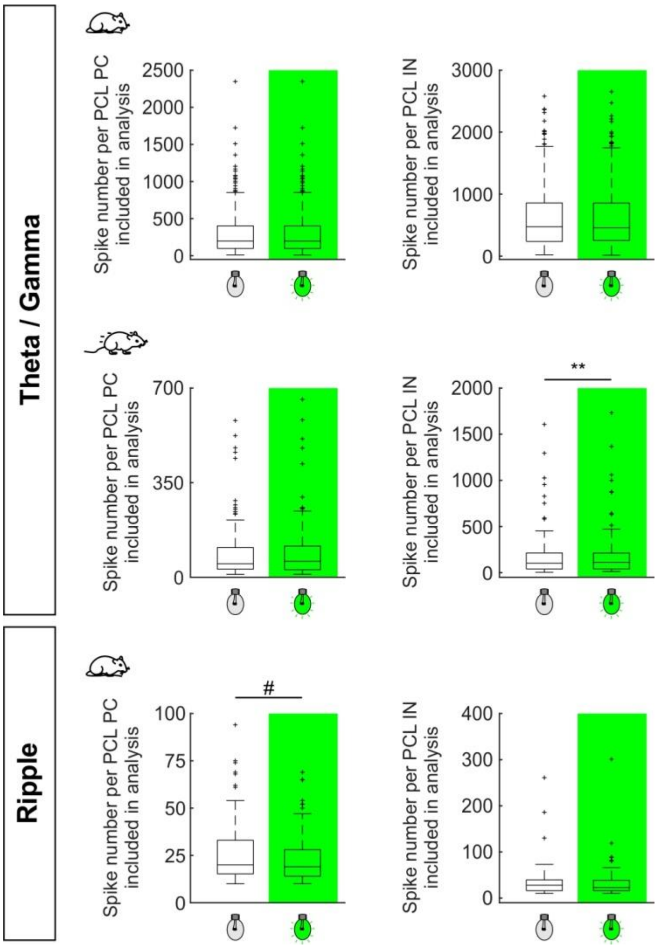

Supplementary Fig. S7 | Number of spikes per single unit included in the spike-phase coupling analysis. Box plots summarizing the spike numbers per CA1 PC (left) and PCL IN (right) included in the spike-theta/gamma- (top) and spike-ripple phase coupling (bottom) in resting (sitting mouse) and running (running mouse) animals. Comparison of the spike numbers for *light off* and *light on* periods (green boxes). Only in the analysis of spike-ripple phase coupling in CA1 PCs and in the analysis of spike-theta/gamma phase coupling in PCL INs in running animals the number of analyzed spikes differed significantly between *light off* and *light on* periods (p=0.0008 and p=0.0031, respectively; Wilcoxon signed-rank test).

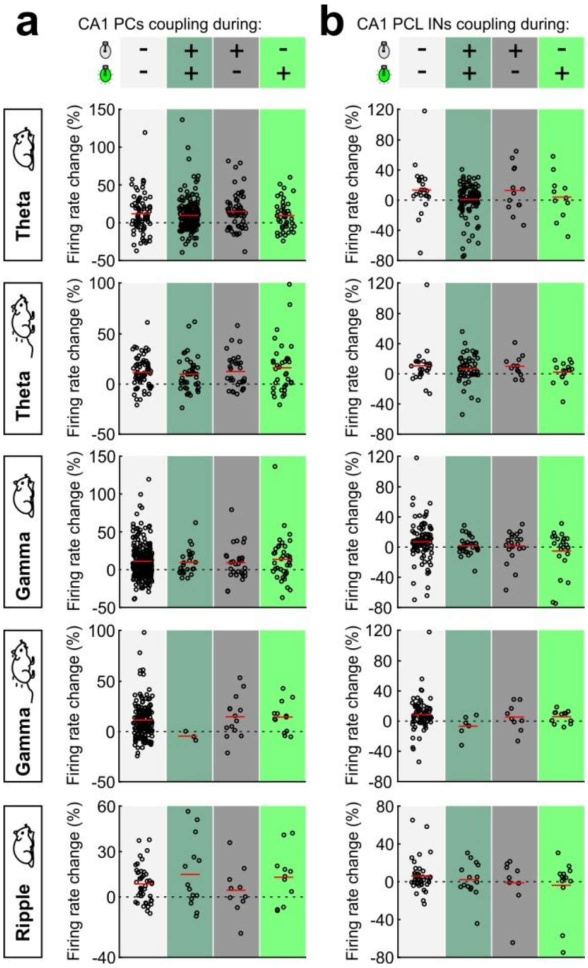

Supplementary Fig. S8 | Correlation of the effect of optogenetic PVI silencing on the average firing rate with the effect on spike-phase coupling. a, b Putative CA1 PCs (**a**) and PCL INs (**b**) were categorized in cells significantly coupling their action potential firing to the phase of ongoing network oscillations neither during light on nor light off periods (grey area, left), during both light on and off periods (dark green area), during light off periods only (dark grey area) or during light on periods only (green area, right). Spike phase coupling was determined for the following conditions (from top to bottom): spike-phase coupling relative to theta oscillations in resting animals; relative to theta oscillations in running animals; gamma oscillations in resting animals; gamma oscillations in running animals; ripple oscillations in resting animals. Single circles represent individual cells. For every cell, the average firing rate change by light stimulation is shown. Red horizontal lines indicate the mean light-mediated firing rate change for the respective cell group at the respective condition. Note that apart from a stronger disinhibition of PCL INs never coupling to theta oscillations in resting mice compared to PCL INs phase coupling to theta oscillations both during *light on* and *light off* periods, there was no correlation between the effect of PVI silencing on the analyzed spike-phase coupling and the effect on the average activity level. PCL, pyramidal cell layer. *, $p<0.05$.
